# Sarcopenia in the foot on magnetic resonance imaging in patients with diabetes mellitus – a systematic review

**DOI:** 10.1186/s40842-024-00194-5

**Published:** 2024-10-25

**Authors:** Manal Ahmad, Abdulla Mohamed, Dimitri Amiras, Francesca Siracusa, Joseph Shalhoub, Alun Huw Davies

**Affiliations:** 1 Section of Vascular Surgery, Department of Surgery and Cancer, Imperial College London, 4th Floor, North Wing, Charing Cross Hospital, Fulham Palace Road, London, United Kingdom; 2 Imperial Vascular Unit, Imperial College Healthcare NHS Trust, London, United Kingdom; 3 Department of Radiology, Imperial College Healthcare NHS Trust, London, United Kingdom; 4 Arabian Gulf University, Manama, Bahrain

**Keywords:** Magnetic resonance imaging, MRI, Diabetes, Diabetic foot disease, Sarcopenia, Lower limb, Foot

## Abstract

**Introduction:**

Sarcopenia is defined by low measures of muscle quantity, quality and reduced physical performance. It is associated with higher levels of frailty. Individuals with diabetes mellitus (DM) undergo sarcopenia at an accelerated rate resulting in structural changes potentially culminating in limb loss.

**Aims:**

To review the evidence on methods of detecting and measuring sarcopenic changes on magnetic resonance imaging (MRI) in the foot in patients with diabetes.

**Methods:**

A literature review was conducted in accordance with PRISMA guidelines. We searched Embase and Medline (via Ovid), CINAHL (via Ebsco Host), Web of Science and Scopus as well as the grey literature. The MeSH terms “sarcopenia” AND “diabetes mellitus” AND “magnetic resonance imaging” were employed in the primary search string.

**Results:**

874 studies were identified. 404 articles were excluded in the title and abstract screening. 33 studies were assessed for eligibility after abstract and title screening was completed by two reviewers. 7 studies evaluating sarcopenia in the foot were included in the final review.

**Conclusion:**

Sarcopenic changes are evident on MRI of the foot in patients with diabetes and is profound in patients with diabetic neuropathy. The general extent and severity of sarcopenia seems to correlate with clinical scores to assess neuropathy and is implicated in the development of diabetic foot disease.

**Supplementary Information:**

The online version contains supplementary material available at 10.1186/s40842-024-00194-5.

## Introduction

The burden of diabetes and its complications is evident the exponential rise in the number of individuals living with diabetes. It is estimated that there are over 4.3 million individuals in the United Kingdom living with diabetes, an estimated 850,000 undiagnosed individuals and another 2.4 million at risk of developing diabetes [1]. The United States of America has 37 million diagnosed cases of diabetes mellitus and 96 million pre-diabetic individuals, and it is estimated that over 537 million people are affected globally [2, 3]. The complex interplay between genetics, obesity, unhealthy diets and sedentary lifestyles has further led to an exponential rise in the number of individuals living with diabetes and it is estimated that the global prevalence of diabetes is expected to reach almost 600 million [4].

Sarcopenia is defined as the ‘age-related’ progressive and global loss of skeletal muscle mass and function. The term was first coined by Rosenberg in 1988 before being superseded by Baumgartner’s definition a decade later, which included a potential method of measuring sarcopenia using height-adjusted appendicular lean mass [5]. The link between inflammatory conditions and sarcopenia is now coming to light [6]. Diabetes is connected to low grade systemic inflammation within the body and persistently elevated levels of glycated haemoglobin is associated with accelerated sarcopenia [7]. The current classifications of sarcopenia are based on a range of anthropomorphic features and physical tests assessing muscle strength, muscle quantity and quality and physical performance. These definitions result from a group of collaborative consensus from the European Working Group on Sarcopenia in older people (EWGSOP2) 2019, The Asian Working Group for Sarcopenia (AWGS) 2019, the International Working Group on Sarcopenia and the American Foundation for the National Institute of Health [6, 8]. More recently, this has been recognised as a muscular disease in the updated International Classification of Diseases (ICD) 10th edition, which will further help to formalise its diagnosis in the aging population [9]. The global economic impact from sarcopenia is significant, costing an additional estimated $19 billion in the United States and £2 billion in the United Kingdom [10]. There is an established bi-directional relationship between diabetes and sarcopenia, with the risk of sarcopenia almost twice as high when compared to their normoglycemic counterparts. This adversely impacts mobility, general functional and cognitive function over time [7, 11]. Accelerated sarcopenia has also been implicated with age, visceral adiposity, albuminuria, osteoporosis, nephropathy and higher C-reactive protein (CRP) [12, 13].

Magnetic resonance imaging (MRI) is a useful imaging modality conferring benefits over other imaging techniques. It provides detailed qualitative information about the quality of the musculature and bone and quantitative information including measurable details pertaining to the soft tissue characteristics and changes and the anatomy [14]. It is the current gold standard imaging modality for soft tissue pathologies and osteall changes such as osteomyelitis and is regularly used in the management of diabetic foot disease [15, 16]. Sarcopenic changes can lead to alteration in the overall structure and biomechanics of the foot.

## Rationale

Sarcopenia is not regularly reported but with increased recognition of sarcopenia as a pathological process which can potentially impact patient mobility in the long term merits the discussion and evaluation on current methods of measuring and reporting sarcopenia in the foot in patients with diabetes and its associated diabetic foot disease related complications.

## Aims and objectives

The aim of this review was to identify the current methods of identifying and measuring sarcopenia in the foot on magnetic resonance imaging (MRI) in patients with diabetes.

## Methods

### Search strategy

The systematic review was registered on PROSPERO (CRD number: 42022379106). It was performed and is reported in accordance with the Preferred Reporting for Systematic Reviews and Meta-Analyses (PRISMA) statement [17]. The existing literature was searched using Embase and Medline (via Ovid interface), CINAHL (via Ebsco Host), Web of Science, and Scopus. This was undertaken on the 24th November 2023. The grey literature was also reviewed on ClinicalTrials.gov, WHO international clinical trials registry platform, Cochrane Library and the NIHR journals library and ProQuest.

The MeSH terms “sarcopenia” AND “diabetes mellitus” AND “magnetic resonance imaging” AND “lower limb” were employed in the primary search string. The complete list of search terms and search strategy is included in appendix 1.

### Eligibility criteria

The population comprised of patients with a diagnosis of Type 1 or Type 2 Diabetes Mellitus with or without diabetic neuropathy or ulceration, who had an MRI of their lower limbs. No time limitation placed on the reviews. The inclusion criteria consisted of articles up to 2023, in all languages, human studies, randomised controlled trials, case–control studies, cohort studies, theses and review articles. Animal model studies, posters, abstracts, commentaries, book chapters, case reports, editorials and expert opinions were excluded.

### Selection process

The results were reviewed on Covidence Software© by two independent reviewers (MA and AM) where the initial abstract screening and full article reviews were conducted. Any discrepancies on article inclusion were discussed by the two reviewers and any disagreements by discussing with a third reviewer to reach a final consensus.

### Risk of bias

The Newcastle–Ottawa scoring system was used to assess the risk of bias for each study [18]. This was done by two individuals.

### Data collection process

Data for the systematic review was collected using a Microsoft Excel spreadsheet by two reviewers.

### Data items

The extracted information included the author, journal, year of publication, patient demographic data, duration of diabetes, BMI of the diabetic patients, presence or absence of ulceration, pre-existing diabetic polyneuropathy or diagnosis of peripheral arterial disease, muscles reviewed, method of measurement, findings, results, statistical analysis, and inter- and intra-rater reliability, where this was reported.

## Results

Eight hundred seventy-four studies were identified from the databases and registers. 843 studies were screened after removal of duplicates. 404 articles were excluded in the title and abstract screening. 439 were sought for retrieval. 33 studies were assessed for eligibility in the full text review of which 13 were excluded due to the incorrect population, outcome, study design, comparator, poster or other reasons. 11 studies assessed the incorrect anatomical regions of the lower limb i.e. quadriceps, rectus femoris, biceps femoris, etc. and were therefore excluded.

**Fig. 1:**
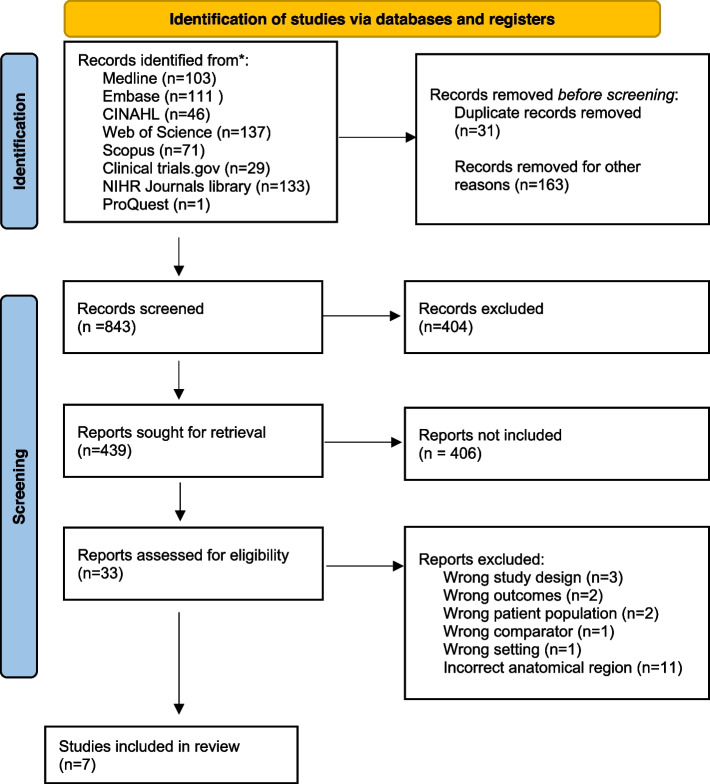
PRISMA Flow Diagram

Seven studies evaluating sarcopenia in the foot were included in the final review (Fig. 1). The Newcastle–Ottawa scale was used to assess risk of bias with the majority of studies graded as good or very good (Appendix 2).

### Patient characteristics

The included studies are summarised in Tables 1 and 2. The studies included spanned from 2002 to 2022 with 7 case-controls and a single cross-sectional study. A total of 234 patients (129 male; 110 female) were included of which 154 patients (approximately 68.5%) had diabetes. Two studies included patients with foot deformities (claw-toe/hammer toe) [19, 20]. A single study included patients with recent ulceration but none of the patients included in the studies had active ulceration at the time of their MRI scan [21]. 5 studies included patients with peripheral neuropathy. The most commonly measured muscles were the intrinsic muscles of the foot whilst a single study measured the abductor hallucis muscle and another also included the extensor digitorum longus and the flexor digitorum longus.

**Table 1:** Summary of studies included

Author	Year	Study Type	Number of patients	Number of patients with Diabetes	Number of diabetic patients with neuropathy	Sex	Age (Range) of DM patients	BMI
Andersen	2004	cross-sectional	46	23 (Type 1 Diabetes)	15	16 M:7F	46 (27–62)	73 kg (53–92)
Andreassen	2009	case control	26	12	4	12 M:8F	56 (45–76)	73 kg (54–100)
Bus	2002	case control	18	8	8	6 M:2F	51.6 ± 11.1	87.8 ± 16.2
Bus	2009	cross-sectional	20	20	10 (+ Claw Deformity)	12 M:8F	58.5 ± 7.1 (claw toe group)	27.3 ± 3.2 (Claw toe group)
Cheuy	2013	case control	19	12	NOS	9 M:10F	57 ± 12 in the diabetic polyneuropathy group	114 kg (21)
Greenman	2005	case control	33	21	12	14 M:5F	56.7 ± 8.38 (non-neuropathic); 51 ± 7(neuropathic)	32 ± 6 (non-neuropathic group)33 ± 4.7 (neuropathic group)
Lue	2022	case control	102	63	NOS	60 M:42F	55.3 ± 10.5	33.1 ± 9.5

**Table 2:** Summary of findings

Author	Year	Muscles Measured	Method of Measurement	Control	Diabetic (Non-Neuropathic)	Diabetic (Neuropathy)
Andersen	2004	Intrinsic muscles of the foot	Cross sectional area (CSA)—Stereological total volume	168 ± 42 cm3	165 ± 34 cm3	86 ± 52 cm3
Andreassen	2009	Intrinsic muscles of the foot	Cross sectional area (CSA)—Stereological total volume	Annual loss of muscle volume: 0.2% (-2.5 to2.4)	Annual loss of muscle volume: -1.1% (-4 to-0.2, p < 0.001)	Annual loss of muscle volume: -2.0% (-4 to-0.2, p < 0.001)
Bus	2002	Intrinsic muscles of the foot	Compositional analysis of the soft tissue (pixel by pixel parametric T 2 colour maps) to measure the cross section area	30.8% [SD ± 3.9%] (range 24.4 –36.8)	NOS	8.3% [SD ± 2.9%] (range 3.7–11.8)
Bus	2009	Intrinsic muscles of the foot, EDL, FDL	5 point atrophy score	NOS	2.6 ± 1.2 (non-deformity group)	3.1 ± 1.1(deformity group)
Cheuy	2013	Intrinsic muscles of the foot	Segmentation and volume measurement	NOS	NOS	NOS
Greenman	2005	Intrinsic muscles of the foot	Ratio of muscle:total area of the foot	0.55 [SD ± 0.04] p < 0.0001	0.44 [SD ± 0.05] p < 0.0001	0.06 [SD ± 0.06] p < 0.0001
Lue	2022	Abductor Hallucis	Goutallier Grading (Fatty Infiltration)	None 85.7% / Diffuse 11.9% / Patchy 2.4%	NOS	None 22.1% / Diffuse 70.6% / Patchy 7.4%

### Methods of measuring sarcopenia in the foot

The primary methods of measurement included stereological total volume measurement, soft tissue compositional analysis, 5-point atrophy scale, segmentation based volumetric measurements and Goutallier grading.

**Fig. 2:**
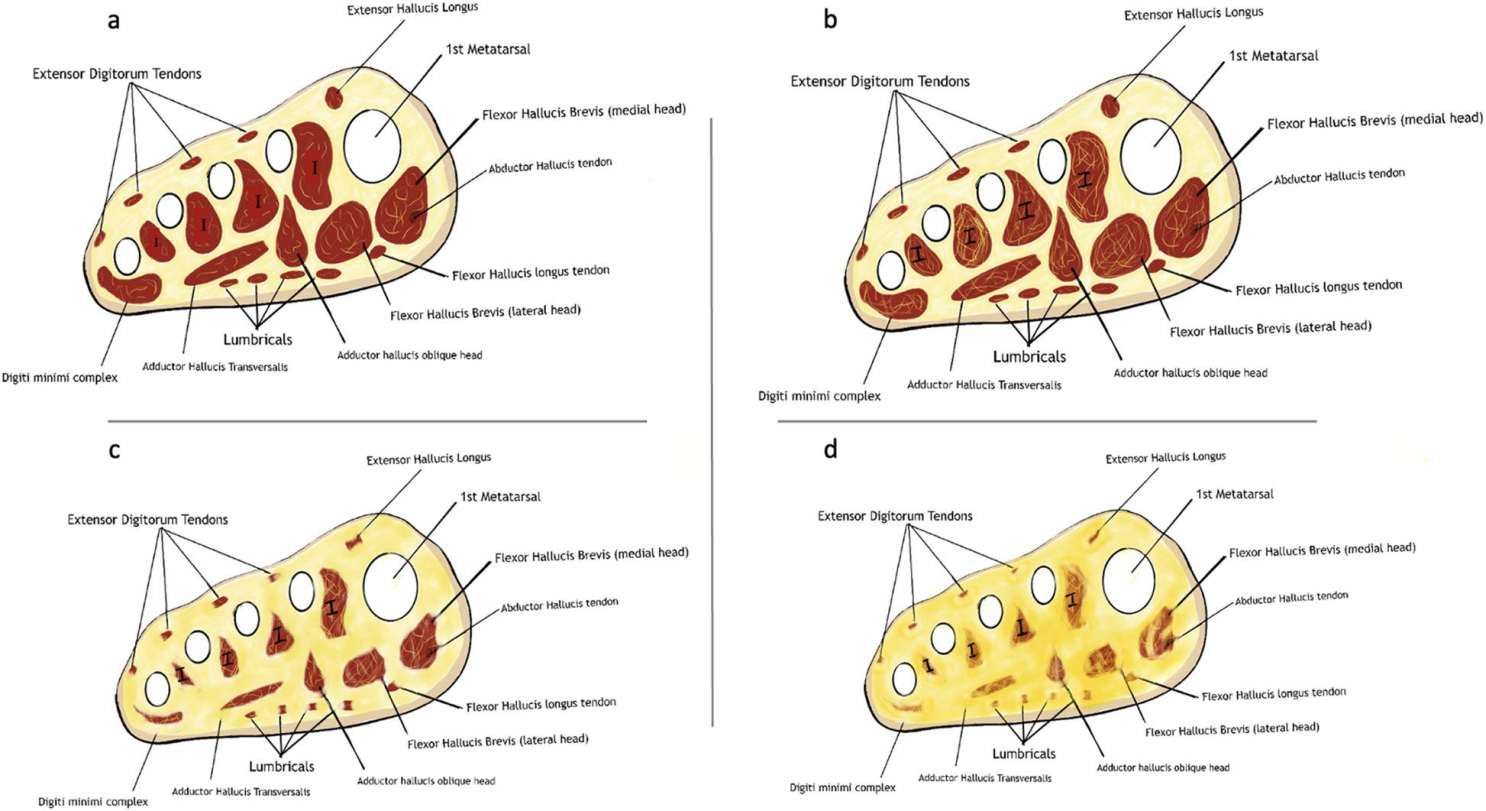
**a**-**d** Schematic representation of gradual fatty infiltration and sarcopenic changes in the forefoot muscles

The included studies reported several methods of measurement. The underlying principles are based on differentiating fat from the surrounding musculature and establish a ratio. In general, the healthy muscle initially demonstrates minor streaks of fat before becoming extensively fibrosed and replaced by fat (Fig. 2a-d). The reported methods of measurement include:

1. **Stereological total volume measurement**

Stereology is a branch of morphometry which allows acquisition of three-dimensional data from two-dimensional sections of any given structure by evaluating small sections of an area of interest called “slices” or “slabs” [22]. It utilises Cavalieri’s principle to allow volume estimation of a structure. The area of interest of interest is divided into serial sections or slabs and the volume of each slab is estimated obtaining the surface area of every section and the section thickness and multiplying the two values to give an unbiased estimate of the volume of a given structure [22]. This is often used in histology or biochemistry but is also used in radiology and MRI to measure cartilage thickness, cardiac function and breast volume [23–25]. Andersen et al. utilised this method for the foot and also used in subsequent studies by the same group [26, 27]. This is whereby the area of interest is intersected by a series of equidistant parallel lines to create a systematic array of test points placed at random on the MR images. The cross-sectional area of the muscle is then calculated at predetermined levels by multiplying the number of muscle tissue points over a certain distance by the unit test point area [26]. Following this, the cross-sectional area measurement is multiplied by T to give a muscle volume estimate.

2. **Soft tissue compositional analysis**

Profoundly sarcopenic patients have a lack of clear muscle delineation which makes direct measurements challenging. To address this, Bus et al. used pixel based parametric T2 colour maps based on echo spin times with a threshold cut-off to visualise the musculature and allow segmentation. The muscle cross-sectional area is expressed as a percentage of the total foot cross sectional area [20, 28, 29]. Further analysis using the segmentation analysis is verified through visual inspection and the T2 colour maps [20].

3. **5-Point atrophy scale**

Proposed by Bus et al., the 5-point atrophy scale is a visual scale representing the extent of sarcopenia. 0 is indicative of healthy muscle tissue, 1–3 indicative of mild, moderate and severe atrophy and 4 representing complete or almost-complete loss of muscle [19, 29]. This measurement has been demonstrated to be reliable with a kappa weight of 0.94 and has been used in subsequent studies for measurements by the same group [19, 29, 30].

4. **Segmentation-based volume measurements**

This method involves segmentation of muscles by rater(s) using two-dimensional renditions of the MRI scans by selecting certain slices at the relevant points of interest, i.e. forefoot, midfoot, hindfoot. The voxel, a single data point in a three-dimensional grid, renders the general intensity within that part of the MRI image. This is then used to create a histogram of the signal intensities and threshold cut-offs are used to differentiate muscle from fat. The method is similar to soft-tissue compositional analysis with the main difference being the method in which the histogram signal intensities are established.

5. **Goutallier Grading**

The Goutallier grading was initially proposed by Goutallier et al. for measuring fatty muscle degeneration on computed tomography in rotator cuff injuries [31]. This was classified from grade 0,indicative of normal muscle, to grade 4,where > 50% muscle is atrophied and infiltrated by fat. In the last decade, this has also been attempted with MRI although there is a lack of consensus on the validation of this classification. Significant variation in the inter- and intra-rater reliability has been noted based on the expertise, i.e. radiologists versus orthopaedists, but not on the duration of experience or the frequency of reporting [32, 33].

### Clinical correlation

The included studies found a significant reduction in the volume of muscle and greater fatty infiltration within the muscle when comparing controls to non-neuropathic diabetic patients with sarcopenic changes. This was more pronounced when comparing control to neuropathic patients with diabetes and is summarised in Table 2 [20, 21, 26, 29, 34–36]. The severity of sarcopenia on imaging also correlated with findings from clinical tests including neuropathy disability scores, vibration perception and monofilament tests [21, 26]. Meaningful statistical analysis of the results could not be undertaken due to the significant heterogeneity in the measurement methods.

## Discussion

**Fig. 3:**
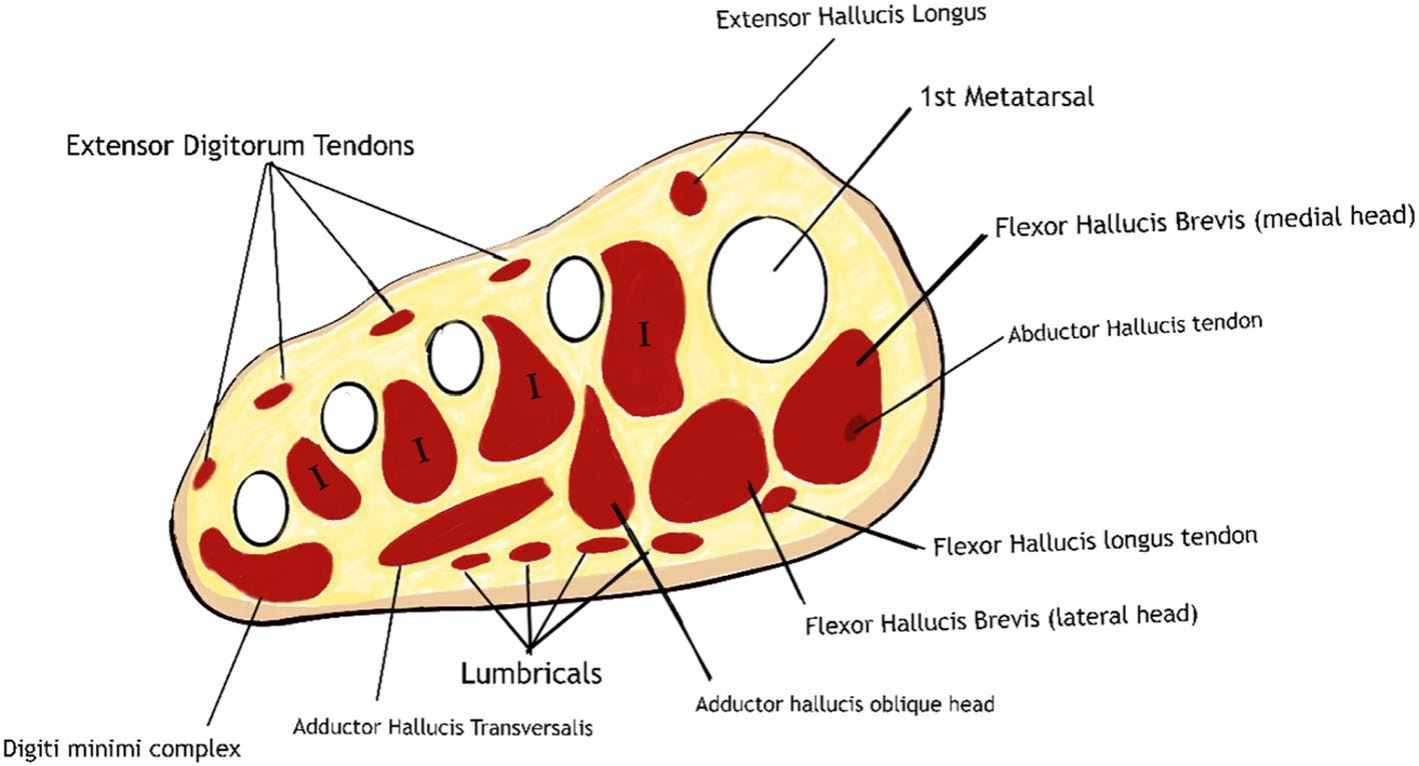
Schematic representation of cross-sectional MRI of the forefoot in healthy individuals

The structure of the foot is comprised of extrinsic and intrinsic muscles which work synergistically to aid in stability and mobility (Tables 3 and 4). The extrinsic muscles are within the foot and ankle are primarily involved in plantar and dorsiflexion as well as inversion and eversion of the foot and ankle. The intrinsic muscles of the foot have their origins and insertion within the foot and provide stability, balance and support to the foot and are almost non-existent in quadrupeds [37, 38]. These are divided into two subgroups – plantar and dorsal, the former of which is further divided into 4 layers and aid with the flexion, extension, adduction and abduction of the toes. A schematic representation of this is presented in Fig. 3. Progressive muscle sarcopenia can culminate in changes to the structure of the foot and can alter the general biomechanics within the foot and can potentially lead to other associated foot deformities including callus formation, claw and hammer toes [29, 38]. The anatomical relation of the intrinsic muscles of the foot with the metatarsal heads further leads to alteration in the areas over which pressure is applied in the foot and can result in ulceration, infection and amputation.

**Table 3:** Extrinsic muscles of the foot and function

	**Muscle**	**Function**
**Anterior Compartment**	Tibialis Anterior	Ankle dorsiflexion and Foot inversion
	Extensor Hallucis Longus	Extension of hallux
	Extensor Digitorum Longus	Extension of 2nd-5th digits
	Peroneus Tertius	Ankle plantarflexion and Foot eversion
**Lateral Compartment**	Peroneus Longus	Ankle plantarflexion and Foot eversion
	Peroneus Brevis	
**Posterior Compartment**	**Deep Muscles**	
	Tibialis Posterior	Foot inversion, plantarflexion and adduction
	Flexor Hallucis Longus	Flexion of hallux
	Flexor Digitorum Longus	Flexion of 2nd-5th digits
	**Superficial Muscles**	
	Gastrocnemius	Ankle plantarflexion
	Soleus	
	Plantaris

**Table 4:** Intrinsic muscles of the foot and function

	**Muscle**	**Function**
**Plantar Group**		
*** 1st Layer (superficial)***	Abductor Hallucis	Abduction and Flexion of Hallux
	Abductor Digiti Minimi	Abduction of the 5th Digit
	Flexor Digitorum Brevis	Flexion of 2nd-5th Digits
*** 2nd Layer***	Lumbricals	Flexion of the metatarsophalangeal joints and extension at the interphalangeal joints
	Quadratus plantae	Flexion of 2nd-5th Digits
*** 3rd Layer***	Adductor Hallucis	Adduction of the Hallux at the metatarsophalangeal joint
	Flexor Hallucis Brevis	Flexion of the Hallux at the metatarsophangeal joint
	Flexor Digiti Minimi Brevis	Flexion of the 5th Digit at the metatarsophalangeal joint
*** 4th Layer (deep)***	Plantar Interossei	Adduction of 3rd-5th Digits
	Dorsal Interossei	Adduction of 2nd-5th Digits
**Dorsal Group**		
	Extensor Hallucis Brevis	Extension of Hallux
	Extensor Digitorum Brevis	Extension of 2nd-4th Digits

In healthy individuals, approximately 80% of the glucose uptake occurs in skeletal muscle. In diabetes, however, this process is impaired and leads to muscle atrophy, fibre-type transition, fatty acid oxidation and altered myokine secretion. This results in the eventual intramuscular fat deposition within the muscle [39, 40]. Glycation end products, which are implicated in microangiopathy, neuropathy and muscle changes, lead to altered biomechanics within the foot, altered pressure to certain areas, and eventually diabetic foot ulceration and diabetic foot disease [41–43].

Ulcers, when superimposed with infection in immunocompromised patients, can result in osteomyelitis, charcot foot and can progress to diabetic foot sepsis, minor or major amputation and death. Claw toe deformities have been found to be predictive of diabetic foot ulceration [29]. However, Bus et al. found atrophy in the intrinsic muscle were not associated with changes in the angles of the digits. They instead attributed these to alterations in the in the plantar aponeurosis and plantar fat pads [29]. This change in the plantar fat has also been noted in other studies with increased irregularity alongside increased muscle oedema on MRI [44].

The studies included in this review demonstrate increased fatty infiltration in individuals with diabetes (Table 2). The mechanism for this is proposed to be irregular differentiation of the fibroadipogenic progenitor cells into adipocytes as a result of insulin resistance. This results in more intramuscular fat which, in turn, leads to further insulin resistance, creating a cyclical chain of events [36]. Interestingly, the progression of sarcopenic changes seems to begin in the distal peripheries in the intrinsic muscles and, in particular, the interossei in patients with diabetic neuropathy before involving the proximal musculature in the foot [20, 26].

The methods of measuring sarcopenia all have the underlying principle of evaluating either the volume of muscle or the degree of fatty infiltration within the muscle. Stereological muscle measurements are often cumbersome and time consuming, especially when dealing with large amounts of data and is not always a feasible option in clinical practice. The Goutallier grading system has its own controversy in the reliability of its use given the significant variation in the interpretation of severity, particularly when the difference may not be glaringly obvious and lead to difference in its overall interpretation. However, Bus et al. used their own 5-point scale which is not dissimilar to the Goutallier grading system and were able to demonstrate very good inter and intra rater reliability. It is worth noting though that this was primarily used by the same group with the only partially external use of this method of measurement by Chen et al. who worked in collaboration with Bus et al.’s group and is yet to be validated externally [45]. Segmentation and soft tissue compositional analysis is a useful method which allows objective reproducibility and can be time efficient. [20, 35]. A semi-automated method to measure was also utilised which yielded intra-rater correlation co-efficient of > 0.99 for intra-rater measurements and 0.96 for all inter-rater measurements which is excellent [35]. Greenman et al. adapted rapid acquisition with relaxation enhancement (RARE), usually used for H1 MRI, to phosphorus 31 images to measure the ratio of the muscle to whole-foot cross-sectional area and were able to measure muscle atrophy prior to the presence of neuropathic symptoms [21]. This could allow measurement of sarcopenia prior to the development of overt clinical symptoms and may be worth exploring in the future.

The majority of the included studies in our review had single rater measurements without cross-checking and validation of measurements, and are subject to potential bias based on rater experience and speciality (radiology, orthopaedics, vascular, etc.). Furthermore, the included studies have a small sample sizes primarily composed of patients without active ulceration or amputation. This does not reflect the reality of clinical practice, where many patients have active ulceration, and have a history of intervention or amputation.

The assessment of sarcopenia is based on measuring three components:. muscle mass, muscle strength and physical performance. Dual x-ray absorptiometry is the current ‘gold standard’ for measuring appendicular muscle mass within the realm of the diagnostic definition of sarcopenia [46]. Bioimpedance analysers are used to calculate electrical resistance to gauge the body fat mass, although has been shown to overestimate the muscle mass and can be altered by hydration/dehydration and the presence of oedema [10, 47]. MRI can provide more detailed information about the quality and quantity of the muscle in a non-invasive manner which current modalities may fail to capture to the same extent.

MRI is based on the absorption and emission of radiofrequency energy hydrogen nuclei, which are abundant in water and fat, under the influence of an applied external magnetic field [48, 49]. Variations in radiofrequency pulse sequences can differentiate between adipose tissue and fat free mass as well as bound and free water, with adipose tissue being characterised by a short T1 and a long T2 proton relaxation time [50]. MRI offers benefits over other imaging modalities and can be used to assess skeletal muscle volume and assessment of fat infiltration within muscle, thus providing detailed information on muscle quality as well as other features like bone marrow oedema, osteopenia or osteomyelitis. Fatty infiltration and oedema in the muscles is represented by high signals in the muscle on T1-weighted images and high signal on T2 weighted or short tau inversion recovery (STIR) images [51]. MRI does not involve exposure to ionizing radiation, which makes it an ideal modality for follow-up and surveillance purposes. MRI can also be paired with 31-phosphorous magnetic resonance spectroscopy to measure metabolite concentrations and glycosylation which concurrently affect the structures in the foot [29].

MRI also has certain disadvantages including the cost involved and the requirement for specialised, trained staff and specific equipment and software, along with specialist musculoskeletal radiologists. MRI acquisition and interpretation is often time-consuming for patients and staff. The image can be also be subject to motion artefact and can be contraindicated in individuals with pre-existing metal implants or ferromagnetic devices. The studies included in the review are retrospective and only one study had follow-up imaging of patients [27]. Studies with larger sample sizes are required to further explore the role of sarcopenia in patients with diabetes and diabetic foot ulceration. Most studies excluded patients with existing diabetic foot ulcers and peripheral arterial disease, the combination of which is gradually increasing, and is a more accurate reflection of the current patient cohort seen in clinical practice. It is therefore myopic to assess patients without these symptoms or to assess for peripheral arterial disease which can also result in microinfarction and can also contribute to muscle damage. Multiple methods of measuring atrophy and sarcopenia have been attempted, and a unified and validated method to detect and measure would help with future studies on sarcopenia.

The use of MRI as a longitudinal measurement modality for diagnostic and surveillance purposes would also require standardised protocols and pre-defined parameters for the purpose of gaining information on sarcopenia as signal intensity can vary depending on the protocol and scanner. The majority of the studies in the review were done over 10 years ago. MRI techniques and protocols have vastly evolved and improved within this timeframe, allowing better visualisation and measurements which may not have been previously possible. For example, the Dixon technique, based on chemical shift, is gaining popularity as a method to achieve uniform fat suppression and is capable of demonstrating micro and macroscopic fat [52]. Similarly, chemical shift-based water/fat separation based on multipeak fat spectrum models are also increasingly being used to assess fat infiltration and muscle composition [53]. There are techniques looking a quantifiable and translatable MRI with new programmes emerging on the market to allow segmentation and quantification such as Synthetic MRI, XNAT and 3D slicer. Cheuy et al. used a semi-automated programme to measure adipose from lean muscle and compared this to phantom imaging. Their programme was found to have excellent intra- and inter-rater reliability, demonstrating the potential for using semi-automated or automated techniques in regular MRI foot reporting to identify and measure the extent of sarcopenia in certain patient cohorts [19]. Smith et al.’s study using 7 T high field MRI further works on an efficient and improved measurement of foot muscle morphology using a reduced number of slices and to quantify intrinsic foot muscles. Their study found measurement times could be exponentially reduced and could aid in helping to create accurate and reproducible methods to measure sarcopenia [54]. It is possible that sarcopenia measurements could help identify patients at risk of developing diabetic foot disease and allow for surveillance and risk stratification in future.

## Limitations

There is significant variation in the methods by which sarcopenia is measured and the vast majority of the studies had small sample sizes. Further studies could include bigger cohorts to allow more robust measurements. Furthermore, future studies should include patients with or without ulceration, peripheral arterial disease and osteomyelitis which would be reflective of the true patient population encountered in clinical practice.

## Conclusion

Sarcopenic changes are evident on MRI of the foot in patients with diabetes and are profound in patients with diabetic neuropathy. The general extent and severity of sarcopenia correlates with clinical scores to assess neuropathy and is implicated in the development of diabetic foot disease. Further large-scale studies with patients reflective of current clinical patient cohorts with follow-up are needed as is a validated and uniform method of measuring sarcopenia.

## Registration and protocol

This systematic review was registered on PROSPERO (CRD number: 42022379106) and the protocol is available to view. No amendments were made to this.

## Supplementary Information

Supplementary Material 1.

## Data Availability

Review of existing studies. Further data collected available on request.
