# Supplementary material for: Sarcopenia in the foot on magnetic resonance imaging in patients with diabetes mellitus – a systematic review
Source: Clin Diabetes Endocrinol. 2024 Oct 25;10:31. doi: 10.1186/s40842-024-00194-5 (PMC11515346; doi:10.1186/s40842-024-00194-5)
Supplement: Supplementary file 1 — Supplementary Material 1. [file 40842_2024_194_MOESM1_ESM.docx]

**Appendices**

1. **Search strategy for Ovid Medline**
2. **Search strategy for Ovid EMBASE**
3. **Newcastle-Ottawa grading of studies**

**Search strategy for Ovid Medline**

| # | Query | Results |
| --- | --- | --- |
| 1 | exp Sarcopenia/ or exp Muscular Atrophy/ | 20,354 |
| 2 | myosteatosis.mp. | 328 |
| 3 | musc* ?edema.mp. | 330 |
| 4 | Myopathies, Structural, Congenital/ or Muscle, Skeletal/ | 159,989 |
| 5 | myopath*.mp. | 32,046 |
| 6 | exp Diabetes Mellitus, Type 2/ or exp Diabetes Mellitus, Type 1/ or exp Diabetes Mellitus/ | 491,690 |
| 7 | diabet$.mp. | 810,067 |
| 8 | diabet$ neuropath$.mp. | 19,545 |
| 9 | diabet$ polyneuropath$.mp. | 1,347 |
| 10 | exp Foot/ or exp Diabetic Foot/ or exp Skin Ulcer/ | 94,459 |
| 11 | (diabet* adj (foot or feet)).tw. | 10,510 |
| 12 | (diabet* adj3 defect*).tw. | 1,110 |
| 13 | (diabet* adj3 wound*).tw. | 4,658 |
| 14 | Neuropath*.mp. | 168,345 |
| 15 | 8 or 9 or 10 or 11 or 12 or 13 or 14 | 264,775 |
| 16 | 1 or 2 or 3 or 4 or 5 | 198,640 |
| 17 | 6 or 7 | 812,536 |
| 18 | exp magnetic resonance imaging/ or diffusion magnetic resonance imaging/ or echo-planar imaging/ or fluorine-19 magnetic resonance imaging/ or magnetic resonance angiography/ or magnetic resonance imaging, cine/ or multiparametric magnetic resonance imaging/ | 521,160 |
| 19 | exp Magnetic Resonance Spectroscopy/ | 221,169 |
| 20 | MRI.mp. | 305,372 |
| 21 | Image Interpretation, Computer-Assisted/ or Imaging, Three-Dimensional/ or Magnetic Resonance Imaging/ | 555,453 |
| 22 | 18 or 19 or 20 or 21 | 918,979 |
| 23 | 16 and 17 | 8,278 |
| 24 | 22 and 23 | 426 |
| 25 | 15 and 24 | 52 |
| 26 | segmentation.mp. | 49,335 |
| 27 | exp Diagnosis, Computer-Assisted/ or exp Artificial Intelligence/ or exp Machine Learning/ | 232,513 |
| 28 | convolutional neural network.mp. | 14,329 |
| 29 | exp Pattern Recognition, Automated/ or exp Algorithms/ or exp Magnetic Resonance Imaging/ or exp Deep Learning/ or exp Image Interpretation, Computer-Assisted/ or exp Image Processing, Computer-Assisted/ or exp Neural Networks, Computer/ or exp Machine Learning/ | 1,448,641 |
| 30 | recurrent neural network.mp. | 2,370 |
| 31 | exp Natural Language Processing/ | 5,814 |
| 32 | 26 or 27 or 28 or 29 or 30 or 31 | 1,489,492 |
| 33 | 25 and 32 | 43 |

**Search Strategy for Ovid Embase**

| # | Query | Results |
| --- | --- | --- |
| 1 | exp muscle atrophy/ or exp sarcopenia/ | 57,323 |
| 2 | myosteatosis.mp. | 513 |
| 3 | exp edema/ | 374,741 |
| 4 | musc* ?edema.mp. | 658 |
| 5 | exp diabetes mellitus/ | 1,192,999 |
| 6 | exp insulin dependent diabetes mellitus/ | 134,845 |
| 7 | diabet$.mp. | 1,414,092 |
| 8 | diabet$ neuropath$.mp. | 31,813 |
| 9 | exp foot ulcer/ | 5,907 |
| 10 | exp diabetic foot/ | 19,591 |
| 11 | (diabet* adj3 ulcer*).tw. | 11,024 |
| 12 | (diabet* adj3 (foot or feet)).tw. | 17,152 |
| 13 | (diabet* adj3 wound*).tw. | 6,499 |
| 14 | (diabet* adj3 defect*).tw. | 1,553 |
| 15 | Neuropath*.mp. | 378,658 |
| 16 | 1 or 2 or 3 or 4 | 431,101 |
| 17 | 5 or 6 or 7 | 1,420,439 |
| 18 | 9 or 10 or 11 or 12 or 13 or 14 or 15 | 404,342 |
| 19 | exp nuclear magnetic resonance imaging/ | 1,157,768 |
| 20 | exp nuclear magnetic resonance spectroscopy/ | 134,435 |
| 21 | MRI.mp. | 533,456 |
| 22 | image interpretation.mp. or exp image analysis/ | 223,722 |
| 23 | 19 or 20 or 21 or 22 | 1,476,620 |
| 24 | 16 and 17 | 35,679 |
| 25 | 23 and 24 | 3,202 |
| 26 | 18 and 25 | 523 |
| 27 | exp segmentation algorithm/ or exp image segmentation/ | 18,305 |
| 28 | automated reasoning/ or exp artificial intelligence/ | 68,938 |
| 29 | exp machine learning/ or exp algorithm/ or exp artificial intelligence/ | 733,336 |
| 30 | exp convolutional neural network/ | 18,818 |
| 31 | exp deep learning/ | 31,275 |
| 32 | exp machine learning/ | 346,225 |
| 33 | exp artificial neural network/ or exp recurrent neural network/ | 77,131 |
| 34 | exp computer analysis/ or exp natural language processing/ | 134,412 |
| 35 | 22 or 27 or 28 or 29 or 30 or 31 or 32 or 33 or 34 | 1,059,960 |
| 36 | 26 and 35 | 31 |

**Newcastle-Ottawa grading of studies**

| **Study** | **Year** | **Points** | **Grading** |
| --- | --- | --- | --- |
| **Andersen** | 2004 | 7 | Good |
| **Andreassen** | 2009 | 9 | Very Good |
| **Bus** | 2002 | 7 | Good |
| **Bus** | 2009 | 7 | Good |
| **Greenman** | 2005 | 8 | Good |
| **Lue** | 2022 | 7 | Good |
